# The Frequency of *DYT1* (GAG Deletion) Mutation in Primary Dystonia Patients from Iran

**Published:** 2011-04-21

**Authors:** Mohammad Hamid, Mohammad Taghi Akbari, Gholam Ali Shahidi, Zahra Zand

**Affiliations:** 1. Molecular Medicine Division, Biotechnology Research Center, Pasteur Institute of Iran, Tehran, Iran; 2. Medical Genetics Department, Faculty of Medical Sciences, Tarbiat Modares University, Tehran, Iran; 3. Tehran Medical Genetics Laboratory, Tehran, Iran; 4. Neurology Department, Tehran University of Medical Sciences, Hazrat Rasool Hospital, Tehran, Iran; 5. Science and Research Branch, Islamic Azad University, Tehran, Iran.

**Keywords:** Dystonic Disorder, Primary Dystonia, *DYT1*, Deletion Mutation

## Abstract

**Objective:**

To determine the frequency of *DYT1* mutation in Iranian patients affected with primary dystonia.

**Materials and Methods:**

In this study, we investigated 60 patients with primary dystonia who referred to the Tehran Medical Genetics Laboratory (TMGL) to determine the deletional mutation of 904-906 del GAG in the *DYT1* gene. DNA extracted from patients’ peripheral blood was subjected to PCR-sequencing for exon 5 of the *DYT1* gene. The collection of samples was based on random sampling.

**Results:**

The deletional mutation of 904-906 del GAG in the *DYT1* gene (15099 to 15101 based on reference sequence: NG_008049.1) was identified in 11 patients (18.33%). The average age of affected patients with this mutation was 13.64 ± 7.4 years.

**Conclusion:**

It can be concluded that the *DYT1* deletional mutation of 904-906 del GAG has a high frequency in Iranian patients in comparison with other non-Jewish populations. Therefore, this particular mutation may be the main representative of pathogenic *DYT1* gene for a large proportion of Iranian patients with primary dystonia.

## Introduction

In 1897, Barraquer-Roviralta described a patient with generalized dystonia under the term athetosis (1). Dystonia are a group of movement disorders of unknown etiology with involuntary muscle contractions which lead to abnormal repeating and tortuous moves of one or a few parts of the body (2, 3). Moreover, cell bodies of dopaminergic neurons appear to be enlarged in brains of dystonia patients (4). There are two groups of dystonia disorders: primary and secondary. In the primary or early-onset type, inheritance is dominant with low peneterance (30-40%) (5-8). This type of disorder is caused by a mutation in the *DYT1* gene. The *DYT1* gene discovered in 1997 is located on chromosome 9 and encodes a protein termed *Torsin A*. This protein is a member of a superfamily of ATPases which is a DNA-binding protein with particular homology to heat shock proteins. It is expressed in several tissues, in particular in the central nervous system (CNS) in the basal ganglia (substantia nigra, thalamus, globus pallidus) and cerebral cortex. It is also thought to be involved in cellular trafficking of the dopamine transporter and other membrane-bound proteins (9). This gene (also named *TOR1A*) is the only identified gene responsible for type 1 dystonia and the 3 bp deletion (GAG) in exon 5 (15099 to15101 based on reference sequence: NG_008049.1) is the most common causative mutation. The deletion results in the loss of one of a pair of glutamic acid residues near the carboxy terminus in a conserved region of ATP-binding proteins (*Torsin A*) with unclear function (10). This disorder is commonly observed in infancy and adolescence and usually manifests before 26 years of age. Due to lack of any other accompanying neurological abnormalities, this disorder is also called primary torsion dystonia (PTD). In secondary dystonia, the disorder usually takes place as a result of a background disease condition which might be of genetic origin (such as Wilson syndrome) or acquired due to the effects of some drugs. The disorder in most PTD patients begins with involvement of a limb (leg or hand) with gradual expansion to other parts of the body, becoming generalized (10).

Our aim in this study was to determine the frequency of the 3-bp deletion (GAG) in exon 5 of the *DYT1* gene in patients with primary dystonia as the first study in the Iranian population.

## Materials and Methods

### Patients

A total of 60 patients (34 males and 26 females) suspected of *DYT1* who referred to the Tehran Medical Genetics Laboratory (TMGL) were investigated to determine the deletional mutation of 904-906 del GAG in the *DYT1* gene. The Ethics Committee of Pasteur Institute of Iran approved this atudy. A neurologist diagnosed the type of disorder according to the Fahn et al. Dystonia classification criteria (2), which is based on the involvement of different body sites causing twisting, repetitive movements or abnormal postures. Following written informed consent from the patients or their parents, 10 ml of peripheral blood was taken from each individual, collected in EDTA tubes and kept at -20℃. Whole-blood DNA extraction was carried out by the standard method of salting out (11).

### PCR condition

Utilizing the primers listed in table 1, a 205 bp fragment from exon 5 of the *DYT1* gene was amplified as follows:

94℃ for 5 minutes for initial denaturation followed by 32 cycles at 94℃ for 1 minute, 60℃ for 1 minute and 72℃ for 1 minute. Final extension was carried out for 5 minutes at 72℃. The PCR final volume was 62 µl containing the following: 50 mM KCl, 10 mM Tris-HCl at pH=8.3, 50 mM MgCl_2_, 0.2 mM dNTPs, 10 pM of each of the forward and reverse primers, 0.5 unit Tag DNA polymerase (CinnaGen, Iran) and 500-1000 ng genomic DNA. Amplification of the 205 bp was monitored by electrophoresing 10 µl of the PCR product on 1.5% agarose gel, stained with ethidium bromide and visualized by exposing to UV light.

**Table 1:** Oligonucleotide primers used for amplifying the specific fragment for exon 5 of the DYT1 gene


Primer	Sequence	PCR product

DYT1F	5`-CCTGGAATACAAACACCTA-3`	205 bp
DYT1R	5`-GGCTGCCAATCATGACTGTC-3`	


#### Sequence analysis

The sequencing reactions were performed by the chain termination method as described elsewhere (12, 13) by ABI 3730 XL sequencer (Applied Biosystems, Foster City, CA). Amplification for cycle sequencing was conducted utilizing the same forward and reverse primers used for initial amplification of the target gene (Macro Gene, Seoul, Korea).

#### Statistical analysis

Quantitative variables were expressed as means ± SD while qualitative variables were expressed as percentages.

## Results

**Fig 1:**
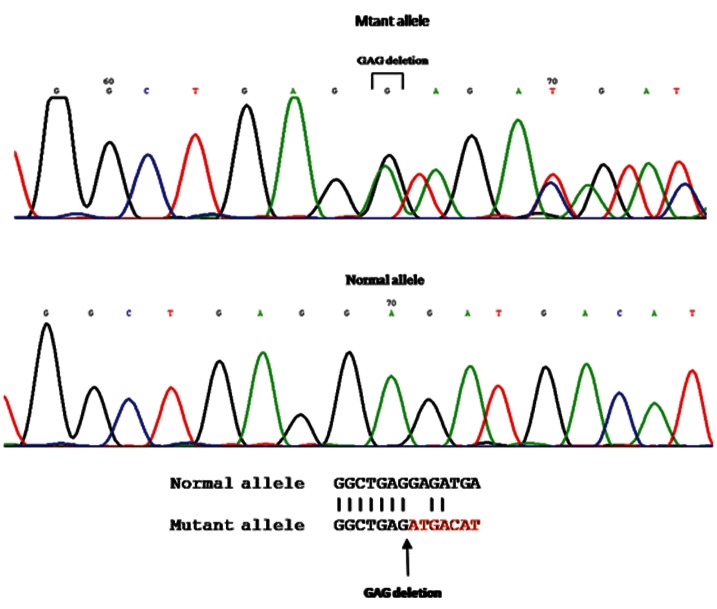
Sequence analysis showing GAG deletion mutation of the DYT1 gene in heterozygous form compared with normal control.

In this study 60 patients suspected of type 1 dystonia were selected and after amplifying the specific fragment for exon 5 of the *DYT1* gene (205 bp) and DNA sequencing, we analyzed the rate of 3 bp GAG deletional mutation (Fig 1).

**Table 2:** Frequency of the GAG deletion mutation in a group of Iranian patients and the mean age at onset of dystonia


Study group	Sex	Number	Mean age in all patients (years ± SD)	Mean age in positive DYT1 patients (years ± SD)	Number (%)

Patients with type 1 dystonia	Male	34	20.4 ± 9.7	11.6 ± 3.4	5 (8.3)
	Female	26	19.65 ± 8.2	15.33 ± 9.6	6 (10)
	Total	60	20.08 ± 9.05	13.64 ± 7.4	11 (18.33)


There were 36 (57%) males and 26 (43%) females. Table 2 summarizes data related to the average age of the patient population and those with positive findings for the mutation. Females (10%) had the highest mutation frequency. The age of the affected patients positive for the GAG deletion mutation was lower compared to the total patient population.

## Discussion

The frequency of the GAG deletion mutation in exon 5 of the *DYT1* gene in a group of Iranian patients with PTD was determined by DNA sequencing. This mutation is the most common cause of type 1 dystonia studied. Its frequency was 18.33% in the studied population (Table 2). This mutation has been reported with 90% and 70% frequency amongst Ashkenazi and non-Ashkenazi Jewish populations, respectively (14). Compared to findings for other non-Jewish populations of European and East Asian origin, the frequency of this mutation in Iranian patients of this study is very high. The frequency of this mutation in different populations summarized in table 3 confirms this conclusion. The only exception, Russian patients (62%), is due to their admixture with the Ashkenazi Jewish population (15). Therefore, the frequency of this *DYT1* deletional mutation derived from this study is different from the non-Jewish population of Europe and East-Asia, and remarkably high. Considering the fact that Jewish communities are closed with regard to marriage with the non-Jewish population, the likelihood of admixture with the Iranian population is very remote. As a result the observed high frequency for the GAG deletion in Iranian patients must have an independent cause and possibly a particular "founder effect" might have been involved. Also, in this study patients with the GAG deletion compared to the total patient population had a lower average age of 13.64 ± 7.4 years versus 20 years. This average age was approximately similar to other populations (16, 17).

Due to technical advances in recent years, interest in functional surgical approaches in dystonia has been renewed, in particular deep brain stimulation (DBS) of the globus pallidus and pallidotomy. Although experience with this approach is still limited, preliminary results in patients with primary generalized dystonia, especially *DYT1* GAG deletion carriers, seem to show better response than patients with other types of dystonia (9, 18). Therefore, DBS may be helpful in selected early onset torsion dystonia patients with severe generalized dystonia.

**Table 3:** Summary of frequency of *DYT1* (GAG deletion) amongst non-Ashkenazi Jewish patients in European and Asian countries


Population	Total patients	%	Positive DYT1 (GAG deletion) patients

Europeans			
French			
(Dhaenens et al., 2005) (21)	104	0	0
Germans			
(Maniak et al., 2003) (22)	89	0	0
(Grundmann et al., 2003) (23)	45	6.67	3
(Kamm et al., 2000) (24)	37	0	0
(Kamm et al., 1999) (25)	45	6.67	3
Italian			
(Zorzi et al., 2002) (26)	27	7.41	2
Serbian			
(Major et al., 2001) (27)	34	2.94	1
Danish			
(Hjermind et al., 2002)(28)	103	0.97	1
Asian			
Japanese			
(Matsumoto et al., 2001) (29)	159	0.62	1
South-west Chinese			
(Zhang et al 2010) (30)	71	1.4	1
Taiwanese			
(Lin et al., 2006) (31)	189	1.5	3
South Korean			
(Im et al 2004) (32)	162	3.1	5
Singaporeans			
(Jamora et al, 2006) (33)	54	0	0
Iranian			
This study	60	18.33	11


## Conclusion

The high frequency of the GAG deletional mutation of *DYT1* in Iranian PTD patients compared to other non-Jewish populations is quite outstanding. Therefore, this mutation is responsible for a significant proportion of affected Iranian patients. For genetic diagnosis of the remainder of the patients, more analyses of other genes implicated in primary dystonia, such as the *DYT6 (THAP1)* gene, are necessary (19, 20).
